# A cluster analysis of chronic obstructive pulmonary disease in dusty areas cohort identified three subgroups

**DOI:** 10.1186/s12890-017-0553-9

**Published:** 2017-12-16

**Authors:** Suhyun Kim, Myoung-Nam Lim, Yoonki Hong, Seon-Sook Han, Seung-Joon Lee, Woo Jin Kim

**Affiliations:** 1 Department of Internal Medicine, Seoul Medical Center, Seoul, Korea; 2 Kangwon National University Data Analytics Center, Chuncheon, Korea; 3 Department of Internal Medicine and Environmental Health Center, Kangwon National University, Chuncheon, Korea

**Keywords:** Chronic pulmonary obstructive disease, Phenotypes, Dusty areas

## Abstract

**Background:**

Chronic obstructive pulmonary disease (COPD) is a heterogeneous disease with variable clinical manifestations, structural changes, and treatment responses. In a cohort study, we performed a baseline cluster analysis to identify the subgroups of COPD and to assess the clinical outcomes of each subgroup during a 1-year follow-up.

**Methods:**

We analyzed dusty areas cohort comprising 272 patients with COPD. The main factors with the highest loading in 15 variables were selected using principal component analysis (PCA) at baseline. The COPD patients were classified by hierarchical cluster analysis using clinical, physiological, and imaging data based on PCA-transformed data. The clinical parameters and outcomes during the 1-year follow-up were evaluated among the subgroups.

**Results:**

PCA revealed that six independent components accounted for 77.3% of variance. Three distinct subgroups were identified through the cluster analysis. Subgroup 1 included younger subjects with fewer symptoms and mild airflow obstruction, and they had fewer exacerbations during the 1-year follow-up. Subgroup 2 comprised subjects with additional symptoms and moderate airflow obstruction, and they most frequently experienced exacerbations requiring hospitalization during the 1-year follow-up. Subgroup 3 included subjects with additional symptoms and mild airflow obstruction; this group had more female patients and a modest frequency of exacerbations requiring hospitalization.

**Conclusions:**

Cluster analysis using the baseline data of a COPD cohort identified three distinct subgroups with different clinical parameters and outcomes. These findings suggest that the identified subgroups represent clinically meaningful subtypes of COPD.

**Electronic supplementary material:**

The online version of this article (10.1186/s12890-017-0553-9) contains supplementary material, which is available to authorized users.

## Background

A recent consensus definition proposed that a chronic obstructive pulmonary disease (COPD) phenotype is “a single or combination of disease attributes that describe differences between individuals with COPD as they relate to clinically meaningful outcomes (symptoms, exacerbations, response to therapy, rate of disease progression, or death)” [1]. COPD heterogeneity has been broadly characterized as an emphysema- and airway-predominant disease, and some of these phenotypes, such as upper lobe-predominant emphysema and the “frequent exacerbator” subtype, have important consequences for clinical management [2–4]. Other factors, including low body mass index (BMI), severity of symptoms, and quality of life, are also important in COPD [1]. Clinical management in accordance with the subtype will improve the outcomes.

We hypothesized that the COPD cohort of Korea comprises discrete groups of subjects with different clinical characteristics associated with different outcomes. To test this hypothesis, we used clustering to identify COPD subgroups and then determined the relationships among pulmonary function decline, exacerbation frequency, and progression of symptoms over 1 year. Some of the results of this study have been previously reported in the form of abstracts [5].

## Methods

### Study design and data collection

**Fig. 1:**
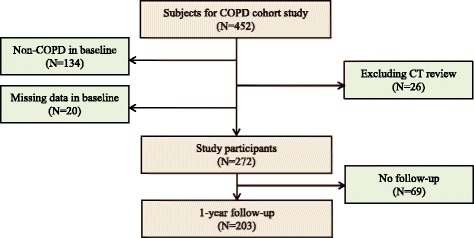
Selection of the study patients from the initial dusty areas cohort study

Data from a cohort comprising 272 patients diagnosed with COPD, who were residing in dusty areas in Korea, were analyzed. Patients were selected from a Korean COPD cohort, which was developed to observe the longitudinal outcomes of COPD subjects living near cement plants. The methods for recruiting patients with COPD in dusty areas (CODA) cohort have been published previously [6]. Briefly, the inclusion criteria for COPD were age > 40 years and post-salbutamol forced expiratory volume in 1 s/forced vital capacity (FEV1/FVC) <0.7. We excluded subjects with bronchiectasis and lung damage caused by tuberculosis. Initially, 272 patients were selected from 452 subjects living near cement plants, and 203 patients who completed the 1-year follow-up were included for outcome analysis (Fig. 1). All patients were evaluated at enrollment using a medical interview, physical examination, spirometry, laboratory tests, and computed tomography (CT) scan. The initial questionnaire included demographics, disease history, residence location, environmental exposure, and self-reported exacerbation history. Exacerbations were defined as follows: hospitalization with systemic steroids and/or antibiotics due to worsening symptoms (dyspnea, cough, or sputum) or medication change with steroid medication and/or antibiotics at the outpatient clinic [7]. The intensity and duration of respiratory symptoms, such as cough, sputum, dyspnea, and wheezing, were evaluated. Dyspnea was evaluated using the modified Medical Research Council Dyspnea (mMRC) scale. Health-related quality of life was evaluated by calculating the total score on the patient-reported COPD assessment test (CAT). Patients were questioned regarding their history of direct exposure to biomass using the following question: “For cooking and/or heating, have you ever been exposed to fuels such as wood and charcoal?” Positive exposure to biomass was defined as direct exposure for 10 years.

The volumetric CT scans were performed using a method reported in previous studies [7, 8], as summarized below. Volumetric CT scans were taken at full inspiration and expiration using a first-generation dual-source CT system (Somatom Definition, Siemens Healthcare, Forchheim, Germany). Whole-lung images were extracted automatically using in-house software, and the attenuation coefficient of each pixel was calculated. From the CT data, the volume fraction (%) of the lung below −950 Hounsfield Unit at full inspiration was calculated automatically (emphysema index, EI). The ratio of mean lung density on expiration and inspiration was calculated. Airway dimensions, including wall area (WA), lumen area, and WA% (ie, WA/[WA + lumen area] × 100), were measured near the origin of the right apical and left apico-posterior segmental bronchi. To identify the extent of emphysema and changes in airway disease quantitatively, we employed the most frequently used methods, the EI for emphysema extent, and WA% to assess airway disease [9, 10].

This study adhered to the clinical practice guidelines and tenets of the Declaration of Helsinki. It has been approved by the Institutional Review Board of Kangwon National University Hospital (KNUH) 2012–06-007. All participants provided written informed consent.

Patients were classified into “ABCD” categories according to their respiratory symptoms as per the mMRC and CAT scores and according to their future risk due to either a recent history of COPD exacerbation or predicted FEV1%, as recommended by the Global Initiative for Chronic Obstructive Lung Disease (GOLD) [11].

### Statistical analysis

The main factors with the highest loading in 15 variables were selected using principal component analysis (PCA) at baseline. COPD patients were classified through hierarchical cluster analysis using clinical, physiological, and imaging data based on PCA-transformed data. The clinical parameters and outcomes during the 1-year follow-up were evaluated among the subgroups.

Factor analysis was performed using the following variables: body mass index (BMI), cigarette smoking amount, mMRC score, CAT score, white blood cells (WBCs) with polymorphonuclear neutrophils (PMNs), eosinophils, interleukin (IL)-6, C-reactive protein (CRP), uric acid, EI, FEV1, FVC, and FEV1/FVC. In order to select the number of important factors, we chose values that has a factor loading greater than 0.6 and a eigenvalue was greater than 1. Oblimin method was used in the square rotation. The similarity of data was calculated using the principal factors that were identified by PCA-transformed data. We performed a hierarchical cluster analysis using Ward’s method with squared Euclidean distance based on the similarity of data by factor analysis score. We then compared the baseline characteristics and changes after 1 year in FEV1, mMRC and CAT scores, and exacerbations according to the subgroups derived through the cluster analysis and 2011 GOLD COPD grouping system. Quantitative variables were compared using an analysis of variance model, and qualitative variables were compared using the chi-square test (SAS ver. 9.3, Cary, NC). All analyses were performed with the level of significance set at *p* < 0.05.

## Results

### Factor analysis and cluster analysis for the identification of COPD subgroups

The Kaiser–Meyer–Olkin (KMO) value was 0.593, and the *p*-value of Bartlett’s test of sphericity was <0.001 (*p* = 0.000). KMO index for each variable was more than 0.5 except that index for uric acid was 0.500. The six factors that significantly contributed to explaining the relationships among the 15 variables accounted for 77.3% of the information. The following representative variables were chosen based on relatively high factor loading: pre- and post-bronchodilator FEV1% and FVC% (for factor 1); WBCs with PMNs (for factor 2); mMRC and total CAT scores (for factor 3); CRP and IL-6 values (for factor 4); BMI and EI (for factor 5); and eosinophils, cigarette smoking amount, and uric acid (for factor 6) (Table 1 and Additional file 1: Table S1). After excluding uric acid, the results were similar.

**Table 1:** Correlations of the 15 original variables with the six main factors derived from the factor analysis in the 272 COPD subjects

	Factor 1	Factor 2	Factor 3	Factor 4	Factor 5	Factor 6
Eigenvalue	3.380	2.152	1.654	1.391	1.341	1.164
% variance explained	25.936	14.346	11.028	9.273	8.974	7.762
Pre FVC, %	*.971*	−.052	.072	−.035	−.149	.003
Post FVC, %	*.924*	.079	.029	−.026	−.049	−.009
Pre FEV1, %	*.924*	−.008	−.048	.004	.038	−.029
Post FEV1, %	*.918*	.027	−.048	.009	.097	.003
PMN	.032	*.969*	−.008	.080	−.002	−.064
WBC	.010	*.959*	−.027	−.005	.079	.125
mMRC	.046	−.057	*.936*	.015	.032	.024
CAT total score	−.037	.023	*.908*	.007	−.013	−.023
CRP	−.055	.059	−.015	*.864*	.034	−.098
IL-6	.001	.030	.042	.*820*	−.213	−.054
BMI	−.093	.124	.137	−.081	.*866*	−.014
Emphysema index	−.027	.063	.167	.081	−.*696*	.146
Eosinophils	−.004	.082	.092	−.190	−.037	.*772*
Pack-year	−.093	.037	−.130	−.049	−.292	.*601*
Uric acid	.094	−.149	−.022	.329	.325	.*556*

*FEV*_*1*_ forced expiratory volume in one second, *FVC* forced vital capacity, *PMN* polymorphonuclear neutrophil, *mMRC* modified Medical Research Council Dyspnea Scale, *CAT* COPD Assessment Test, *BMI* body mass index

Significant factor scores are italicized

### Basal characteristics of COPD subgroups

Three distinct subgroups were identified using the cluster analysis (Table 2). Subgroup 1 included the youngest patients, with a mean age of 70.2 years having mild airflow obstruction. Their mMRC and CAT scores were the best, and they had the lowest values of IL-6 and CRP. Subgroup 2 comprised more male patients and had the oldest patients, with a mean age of 76.6 years having more severe airflow obstruction. Their mMRC and CAT scores were the worst, and their pre- and post-bronchodilator FEV1% and FVC% were the most severe among the three clusters. Nearly 90% of these patients had a history of smoking, the BMI values were the lowest, and the IL-6 and CRP values were the highest. In addition, they had the most severe emphysema, as determined from the CT scan. Subgroup 3 comprised more female patients with mild airflow obstruction and modest mMRC and CAT scores. More than 40% of the patients in this group were never-smokers, and their pre- and post-bronchodilator FEV1%, FVC%, and BMI were the best. The extent of emphysema in subgroup 3 was the lowest among the three clusters. However, biomass exposure among the three clusters was the same. In addition, the incidence of comorbidities, including diabetes, cardiovascular, cerebrovascular, and peptic ulcer disease, and the mean WAs of the airways from the volumetric CT scans, were the same among the three clusters. The proportion of bronchodilator responders who showed >200 mL and >12% increases in FEV1 with bronchodilators was approximately 20% in the three clusters.

**Table 2:** Baseline characteristics of 272 COPD subjects according to the three subgroups

	Total	1(*n* = 158)	2(*n* = 48)	3(*n* = 66)	*p*-value^†^
Demographics					
Gender, Male	217(79.8)	129(81.7)	43(89.6)	45(68.2)	0.0129
Age	72.8 ± 7.3	70.2 ± 6.9	76.6 ± 4.8	75.5 ± 6.7	<.0001
Smoking					0.0005
Current	72(26.5)	50(31.6)	13(27.1)	9(13.6)	
Former	128(47.0)	69(43.7)	30(62.5)	29(43.9)	
Never	72(26.5)	39(24.7)	5(10.4)	28(42.4)	
Pack-year	17.8 ± 22.8	18.5 ± 23.3	22.9 ± 22.2	12.7 ± 19.0	0.0468
BMI	23.1 ± 3.2	23.0 ± 2.7	21.9 ± 3.3	24.6 ± 3.3	<.0001
Height	160.1 ± 8.6	160.9 ± 8.5	160. 7 ± 7.8	157.7 ± 9.1	0.0311
Biomass exposure	95(35.5)	62(40.0)	16(34.0)	17(25.8)	0.1254
Physiology					
Stage					<.0001
Mild	147(54.4)	88(55.7)	8(16.7)	52(78.8)	
Moderate	108(39.7)	65(41.1)	29(60.4)	14(21.2)	
Severe	16(5.9)	5(3.2)	11(22.9)	0(0.0)	
Pre FEV1, L	1.72 ± 0.57	1.87 ± 0.54	1.34 ± 0.48	1.82 ± 0.53	<.0001
Pre FEV1, %	75.7 ± 20.7	77.8 ± 18.5	60.9 ± 16.5	88.4 ± 18.2	<.0001
Pre FEV1/FVC	59.8 ± 9.0	61.5 ± 8.2	53.9 ± 9.4	61.4 ± 8.2	<.0001
Post FEV1, L	1.82 ± 0.56	1.98 ± 0.51	1.45 ± 0.48	1.89 ± 0.53	<.0001
Post FEV1, %	79.8 ± 19.7	82.3 ± 16.7	65.9 ± 17.5	91.4 ± 17.3	<.0001
PostFEV1/FVC	58.8 ± 8.4	60.3 ± 7.4	52.8 ± 9.6	61.2 ± 6.1	<.0001
FEV1, reversibility%	52(19.1)	29(18.4)	10(20.8)	13(19.7)	0.9207
Emphysema index(%)	7.7 ± 7.3	6.6 ± 5.3	15.7 ± 10.0	4.1 ± 4.1	<.0001
Mean wall area of Airway(%)	69.4 ± 5.0	69.0 ± 5.3	69.1 ± 4.4	70.0 ± 4.7	0.3535
Symptoms					
mMRC	1.49 ± 1.15	0.85 ± 0.80	2.48 ± 1.01	2.18 ± 1.02	<.0001
0/1/2/3/4	55/112/46/43/16	52/87/13/3/3	2/6/13/21/6	1/19/20/19/7	<.0001
CAT total	17.3 ± 9.6	12.5 ± 7.9	26.3 ± 7.5	21.5 ± 8.4	<.0001
Comorbidities					
Diabetes mellitus	42(15.7)	27(17.4)	8(17.4)	7(10.6)	0.1147
MI	11(4.3)	5(3.3)	1(2.3)	5(7.8)	0.0885
Heart failure	3(1.1)	1(0.7)	1(2.1)	1(1.5)	0.6696
Cerebrovascular	4(1.5)	0(0.0)	2(4.3)	2(3.0)	0.5035
Peptic ulcer	23(8.6)	14(9.0)	4(8.5)	5(7.6)	0.5848
Systemic inflammation index					
IL-6	2.6 ± 3.7	2.0 ± 3.2	4.7 ± 5.1	2.4 ± 2.7	<.0001
IL-8	16.8 ± 17.8	15.4 ± 16.7	20.5 ± 24.7	17.4 ± 14.5	0.2154
CRP	0.3 ± 0.6	0.1 ± 0.2	0.7 ± 1.2	0.2 ± 0.3	<.0001

*mMRC* modified Medical Research Council Dyspnea Scale, *MI* Myocardial infarction

^†^ *p* values correspond to comparisons between the 3 subgroups using Chi-square test or ANOVA, as appropriate

### COPD subgroups according to the GOLD “ABCD” classification

The GOLD “ABCD” categories according to the respiratory symptoms as per the mMRC and CAT scores were different (Table 3). The classification into GOLD “ABCD” categories as per the mMRC scores resulted in most patients being placed in the A group (58.8%, 35.5%, 2.6%, and 1.1% for the A, B, C, and D groups, respectively), whereas as per the CAT scores, most patients were placed in the B group (25.7%, 66.5%, 0.4%, and 7.4%, for the A, B, C, and D groups, respectively). When classified according to the CAT scores, subgroup 1 had less symptoms (39.9% in the A and C groups) and less severe diseases (96.2% in the A and B groups), according to the GOLD classification. Most of the patients (95.8%) in subgroup 2 had additional symptoms (the B and D groups), and 25% had more severe diseases (the C and D groups), according to the GOLD classification. A total of 90.9% of the patients in subgroup 3 had additional symptoms (the B and D groups, especially 86.4% in group B), according to the GOLD classification.

**Table 3:** COPD subgroups according to the GOLD “ABCD” classification

GOLD classifications	Total	subgroup 1 (*n* = 158)	Subgroup 2 (*n* = 48)	subgroup 3 (*n* = 66)	*p*-value†
according to mMRC					<.0001
A	160(58.8)	135(85.4)	5(10.4)	20(30.3)	
B	91(33.5)	17(10.8)	31(64.6)	43(65.2)	
C	7(2.6)	4(2.5)	3(6.3)	0(0.0)	
D	14(1.1)	2(1.3)	9(18.7)	3(4.5)	
according to CAT total score					<.0001
A	70(25.7)	63(39.9)	1(0.2)	6(9.1)	
B	181(66.5)	89(56.3)	35(72.9)	57(86.4)	
C	1(0.4)	0(0.0)	1(2.1)	0(0.0)	
D	20(7.4)	6(3.8)	11(22.9)	3(4.5)	

^†^ *p* values correspond to comparisons between the 3 subgroups using Chi-square test

### Follow-up data of the three COPD subgroups

One-year follow-up data were available for 203 subjects (Additional file 1: Table S2 and Table 4). During the 1-year follow-up, 2.6% of patients in subgroup 1 experienced exacerbations requiring hospitalization, and they exhibited no decline of FEV1. They had few symptoms at the 1-year follow-up; only 30% complained of cough or sputum production, and their mMRC or CAT scores were not aggravated. Patients in subgroup 2 and 3 experienced more frequent exacerbations requiring hospitalization (15.2% and 14.8% respectively). Patients in subgroup 2 showed the most rapid decline of FEV1 (60 mL) although the result was not statistically significant. Patients in subgroup 2 showed the greatest worsening of symptom scores, both in terms of mMRC and CAT scores. Subgroup 3 had modest exacerbations requiring hospitalization (14.8%), which was significantly higher than the other mild disease group (subgroup 1), and modest symptoms, with symptom progression according to the mMRC and CAT scores; however, there was no decline in FEV1.

**Table 4:** The differences between baseline and one-year follow-up among the three subgroups (*n* = 203)

	Total	Subgroup 1 (*n* = 116)	Subgroup 2 (*n* = 33)	Subgroup 3 (*n* = 54)	*p*-value†	Adjusted *p*-value‡
Δ Pre FEV1	−0.01 ± 0.26	0.01 ± 0.24	−0.06 ± 0.32	0.02 ± 0.27	0.3360	0.1520
Δ Post FEV1	−0.01 ± 0.23	−0.002 ± 0.20	−0.05 ± 0.27	0.01 ± 0.25	0.4254	0.2605
Δ mMRC	−0.005 ± 1.001	−0.33 ± 0.98	0.42 ± 0.79	0.39 ± 0.96	<.0001	<.0001
Δ CAT total	1.12 ± 8.10	−0.75 ± 7.92	3.82 ± 7.99	2.89 ± 8.06	0.0021	0.0010
Exacerbation requiring						
Steroid/antibiotic at outpatient clinic	3(1.5)	1(0.9)	1(3.0)	1(1.8)	0.6376	0.4649
Hospitalization	16(7.9)	3(2.6)	5(15.2)	8(14.8)	0.0054	0.1127
Symptoms						
Cough	81(39.9)	35(30.2)	19(57.6)	27(50.0)	0.0037	0.0102
Sputum	76(37.4)	34(29.3)	20(60.6)	22(40.7)	0.0039	0.0028
Chronic Bronchitis	57(28.1)	24(20.7)	16(48.5)	17(31.5)	0.0059	0.0055

^†^ *p* values correspond to comparisons between the 3 subgroups using Chi-square test or ANOVA, as appropriate

^‡^adjusted *p* values correspond to comparisons between the 3 subgroups using Chi-square test or ANOVA with adjusted for sex, age, smoking status and BMI

## Discussion

In this study, we identified three distinct subgroups of COPD through a cluster analysis of 272 patients with CODA cohort. We also demonstrated that the frequency of exacerbations requiring hospitalization, progress of respiratory symptoms, and changes in the mMRC and CAT scores in 1 year varied among these subgroups. Among the three subgroups, subjects with mild COPD were divided into two subgroups (subgroups 1 and 3), according to the number of symptoms. According to the GOLD classification of airflow limitation severity (based on post-bronchodilator FEV1), subgroup 1 (mild disease group) included younger patients who had fewer symptoms, and subgroup 3 (the other mild disease group) included a majority of female patients with more respiratory symptoms. However, subgroup 3 reported more exacerbations requiring hospitalization and more symptom progression during the 1-year follow-up than subgroup 1. Subgroup 2 (moderate disease group) included subjects with additional respiratory symptoms; this group had more frequent exacerbations requiring hospitalization during the 1-year follow up than subgroup 1 did.

There have been several reports on the various phenotypes of COPD in Western countries in order to identify more homogeneous subgroups [12–16]; however, there have been few of such reports from Asian countries [17]. In the Korean Obstructive Lung Disease (KOLD) cohort, three clusters with the following phenotypes were identified: cluster 1 included subjects with moderate-to-severe airflow obstruction and bronchodilator reversibility, cluster 2 included subjects with moderate airflow obstruction without bronchodilator reversibility, and cluster 3 included subjects with severe airflow obstruction without bronchodilator reversibility [17]. In the KOLD cohort, in terms of risk factors, cluster 3 patients showed more severe airflow obstruction and hyperinflation, had greater emphysematous change in the CT scan, and smoked less [17]. Conversely, in the present study, cumulative smoke exposure (pack-years) was the highest in subgroup 2 (moderate disease group with additional symptoms) compared with the milder subgroups. However, the three subgroups of the present study had a similar biomass exposure, which has previously been reported to result in phenotypic differences [18]. Hong et al. suggested that the airway phenotype of COPD was more common in females, and females are more susceptible to the damaging effects of biomass smoke, thereby leading to the development of airway disease [8]. In the present study, subgroup 3 included more female patients, a large number of never-smokers, and a small percentage of emphysema cases; however, they showed no differences in airway wall thickness or biomass smoking history. Cho et al. used clinical and genetic characteristics to cluster patients with COPD in the National Emphysema Treatment Trial Genetics Ancillary Study cohort with severe emphysema: 1) emphysema predominance, 2) milder severity and bronchodilator responsiveness, 3) discordant lung function/CT emphysema and airway severity, and 4) airway predominance [14]. In the present study, subgroup 2 exhibited severe emphysema and the lowest FEV1, and approximately 20% of the patients were bronchodilator responders; this value was not different among the three subgroups. Furthermore, no difference in airway wall thickness was observed among the subgroups. Regarding the 1-year follow-up, an average of 60 mL of FEV1 decline was noted in subgroup 2, but this value was not significantly different among the subgroups. However, the mMRC and CAT scores improved only in subgroup 1, which exhibited fewer symptoms, such as cough, sputum production, and chronic bronchitis, during the 1-year follow-up. The PCA variables were the ones that changed differently according to subgroups. This may have influenced the results. For example, FEV1, was included in the PCA variables, and patient with better lung function showed more lung function decline in the previous report [19]. However, subgroup 2, which have worse lung function showed more decline in the current study. We did not compare the treatment history; therefore, we could not identify which subgroup would benefit from bronchodilators and/or inhaled corticosteroid (ICS) treatment. Lee et al. suggested that the response to long-acting beta2-agonist and ICS treatment varied with the COPD subtype, and the obstruction-dominant COPD patients exhibited the best response compared with the emphysema-dominant patients who had the worst response [20].

Most recently, Castaldi et al. evaluated 10,192 subjects from the COPD Gene cohort: (1) relatively smoking-resistant individuals, (2) individuals with mild upper zone-predominant emphysema and airflow obstruction, (3) individuals with airway-predominant disease, and (4) individuals with severe obstruction and emphysema [4]. These clusters were strongly associated with known COPD-associated variants [21]. The COPD Gene study reported that the severe subgroup had older and more male patients, and the severe emphysema group showed the most frequent exacerbations and the worst symptoms [4]. This was similar to our study in that the subgroup with a relatively severe stage of disease and additional symptoms (subgroup 2) showed the most severe emphysema and the most frequent exacerbations. In addition, this subgroup had more male and relatively older patients, the lowest BMI, and the highest IL-6 and CRP values compared with the mild disease subgroups.

Garcia-Aymerich identified three clusters in 342 patients who were hospitalized for the first time because of an exacerbation of COPD and proposed clinically relevant COPD subtypes [13]. Interestingly, these three clusters relatively correspond to our subgroups in terms of their clinical features and follow-up outcomes, such as subsequent hospitalizations. According to the ‘Phenotype and Course of COPD (PAC-COPD)’ study group [13], one cluster displayed the worst status in most of the respiratory domains of the disease, such as exercise capacity, more frequent hospitalizations due to COPD, and the highest all-cause mortality; these features correspond well with our subgroup 2. The remaining two clusters of the PAC-COPD study group were characterized by a milder respiratory status, which closely resembles our subgroups 1 and 3; one subtype of the milder clusters had a higher prevalence of obesity, cardiovascular disease, diabetes and higher levels of systemic inflammatory markers. In the present study, however, the more severe stage group (subgroup 2) showed the highest IL-6 and CRP values but the lowest BMI, whereas the incidences of comorbidities was not different among the three clusters.

The present study included patients with relatively mild stages of COPD. The mild-severity group was divided into two subgroups according to the symptoms. The subgroup with additional symptoms, among the subjects with mild airway obstruction, experienced more exacerbations, requiring hospitalization during the 1-year follow-up. These findings may provide an important understanding of COPD phenotypes in terms of prognosis of symptoms and may also demonstrate the importance of the early management of COPD [22]. Moreover, the results of the present study suggest that the COPD classification system of the Korean COPD guideline is reasonable for the prediction of disease prognosis in Korean COPD patients. The Korean guideline classified the COPD patients into three groups, combining the GOLD C and D groups into one group (group “da”) [23]. In the present study, the subgroup with additional symptoms and a relatively severe stage of disease (subgroup 2) showed the most frequent exacerbations requiring hospitalization and the most progressive symptoms, according to the mMRC and CAT scores. Subgroup 2 mostly met the criteria for group B; however, 25% of them showed features of groups C and D, according to the GOLD classification. Subgroup 1 included the youngest patients with mild symptoms and mild severity; this group seldom had acute exacerbations or symptom progression. Subgroup 1 mostly met the criteria for group B, but 40% showed group A characteristics, according to the GOLD classification. Subgroup 3 included more female patients with a lower smoking history and showed the mild severity but with more symptoms and acute exacerbations than subgroup 1 did. Subgroup 3 exclusively included group B patients, according to the GOLD classification.

Some potential limitations of our study are as follows. First, Biomass exposure was only measured using self-reported questionnaires and this may explain the lack of differences in biomass exposure among the three subgroups. While biomass exposure may have effects on phenotypic differences of COPD, better measurement of exposure to wood smoke constituents using validated questionnaire instruments or home exposure-monitoring devices will be needed to detect them. Secondly, the study population was localized to several provinces in Korea and the sample size was relatively small. The present study included only patients with mild-to-moderate stage COPD, which might be another limitation. Therefore, our results should be extrapolated to heavy smokers and patients with severe COPD with caution. Thirdly, the population of this study included approximately 20% of bronchodilator responders; however, we did not analyze the proportion of airway disease, as measured by the methacholine reactivity test. Hence, some patients with bronchial asthma or asthma–COPD overlap might be included. However, there were no differences in the percentage of bronchodilator responders and airway thickness among the three subgroups; accordingly, we suggest that there was little confounding due to possible bronchial asthma or asthma–COPD overlap patients. We did not analyze whether the patients used bronchodilators or ICSs; the treatment outcome of the medications was also not analyzed. Lastly, we analyzed only the 1-year follow-up results; therefore, the long-term prognosis, such as mortality, could not be determined. Nevertheless, the possible clinical implication of this study is that our COPD cohort from dusty areas comprises discrete groups of subjects with different clinical characteristics associated with different outcomes as in other COPD cohorts. We identified three subgroups of COPD patients in this population. Although this study included several environmental factors for cluster analysis, additional long-term follow-up and multinational studies using exposure metrics are warranted.

## Conclusions

Three distinct subgroups were identified using a cluster analysis of dusty areas cohort in Korea. Subgroup 1 subjects were younger, and they exhibited fewer symptoms with mild airway obstruction and fewer exacerbations during the 1-year follow-up. Subgroup 2 subjects had moderate airway obstruction, more severe respiratory symptoms, and the most frequent exacerbations requiring hospitalization during the 1-year follow-up. Subgroup 3, which included more female patients, experienced more symptoms, with mild airway obstruction and more frequent exacerbations requiring hospitalization than the other mild disease subgroup (subgroup 1).

## Additional file

Additional file 1: A cluster analysis of chronic obstructive pulmonary disease in dusty areas cohort identified three subgroups. The Additional file 1 contains two additional tables of study data results: **Table S1.** Correlation structure of the variables. **Table S2.** Follow-up demographics of 203 COPD subjects according to the three subgroups. (DOCX 29 kb)

## Abbreviations

BMI: Body mass index
CAT: COPD assessment test
CODA: COPD in dusty areas
COPD: Chronic obstructive pulmonary disease
FEV1: Forced expiratory volume in 1 s
FVC: Forced vital capacity
GOLD: Global Initiative for Chronic Obstructive Lung Disease
ICS: Inhaled corticosteroid
KOLD: Korean Obstructive Lung Disease
mMRC: Modified Medical Research Council Dyspnea scale
PCA: Principal component analysis
PMN: Polymorphonuclear neutrophil
