# Supplementary material for: A cluster analysis of chronic obstructive pulmonary disease in dusty areas cohort identified three subgroups
Source: BMC Pulm Med. 2017 Dec 16;17:209. doi: 10.1186/s12890-017-0553-9 (PMC5732468; doi:10.1186/s12890-017-0553-9)
Supplement: Additional file 1: — A cluster analysis of chronic obstructive pulmonary disease in dusty areas cohort identified three subgroups. The Additional file 1 contains two additional tables of study data results: Table S1. Correlation structure of the variables. Table S2. Follow-up demographics of 203 COPD subjects according to the three subgroups. (DOCX 29 kb) [file 12890_2017_553_MOESM1_ESM.docx]

Supplement table 1. Correlation structure of the variables

|  | Pre FVC, % | Post FVC, % | Pre FEV1, % | Post FEV1, % | PMN | WBC | mMRC | CAT total score | CRP | IL-6 | BMI | Emphysema index | Eosinophils | Pack-year | Uric acid |
| --- | --- | --- | --- | --- | --- | --- | --- | --- | --- | --- | --- | --- | --- | --- | --- |
| Pre FVC, % | 1.000 | 0.834** | 0.827** | 0.782** | -0.023 | -0.056 | -0.080 | -0.169** | -0.133* | -0.144* | 0.022 | -0.151** | -0.098 | -0.173** | 0.005 |
| Post FVC, % |  | 1.000 | 0.758** | 0.939** | -0.001 | -0.002 | -0.172** | -0.242** | -0.120* | -0.134* | 0.094 | -0.334** | -0.131* | -0.192** | 0.059 |
| Pre FEV1, % |  |  | 1.000 | 0.825** | 0.075 | 0.052 | -0.131* | -0.189** | -0.095 | -0.140* | 0.153** | -0.133* | -0.096 | -0.164** | 0.012 |
| Post FEV1, % |  |  |  | 1.000 | 0.030 | 0.034 | -0.180** | -0.236** | -0.113 | -0.133* | 0.150* | -0.345** | -0.106 | -0.191** | 0.107 |
| PMN |  |  |  |  | 1.000 | 0.902** | 0.007 | 0.057 | 0.169** | 0.169** | 0.033 | 0.134* | 0.035 | 0.089 | -0.007 |
| WBC |  |  |  |  |  | 1.000 | -0.016 | 0.041 | 0.136* | 0.111 | 0.073 | 0.066 | 0.236** | 0.123* | 0.065 |
| mMRC |  |  |  |  |  |  | 1.000 | 0.726** | 0.046 | 0.121* | 0.073 | 0.119* | 0.073 | -0.042 | 0.032 |
| CAT total score |  |  |  |  |  |  |  | 1.000 | 0.081 | 0.120* | 0.049 | 0.169** | 0.051 | 0.015 | -0.045 |
| CRP |  |  |  |  |  |  |  |  | 1.000 | 0.504** | 0.004 | 0.103 | 0.025 | -0.003 | 0.199** |
| IL-6 |  |  |  |  |  |  |  |  |  | 1.000 | -0.152* | 0.144* | 0.018 | 0.140* | 0.104 |
| BMI |  |  |  |  |  |  |  |  |  |  | 1.000 | -0.339** | 0.008 | -0.138* | 0.170** |
| Emphysema index |  |  |  |  |  |  |  |  |  |  |  | 1.000 | 0.105 | 0.118* | -0.027 |
| Eosinophils |  |  |  |  |  |  |  |  |  |  |  |  | 1.000 | 0.178** | 0.145* |
| Pack-year |  |  |  |  |  |  |  |  |  |  |  |  |  | 1.000 | 0.092 |
| Uric acid |  |  |  |  |  |  |  |  |  |  |  |  |  |  | 1.000 |

*P<0.05, **p<0.01

FEV_1 ;_ forced expiratory volume in one second, FVC ; forced vital capacity, PMN ; polymorphonuclear neutrophil, mMRC ; modified Medical Research Council Dyspnea Scale, CAT ; COPD Assessment Test, BMI ; body mass index

Supplementary Table 2. Follow-up demographics of 203 COPD subjects according to the three subgroups

|  |  | Total | 1(n=116) | 2(n=33) | 3(n=54) | p-value† |
| --- | --- | --- | --- | --- | --- | --- |
|  | Gender, Male | 168(82.8) | 99(85.3) | 29(87.9) | 40(74.1) | 0.1350 |
|  | Age | 73.6±6.9 | 71.1±6.7 | 77.4±4.6 | 76.7±6.4 | <.0001 |
|  | Smoking |  |  |  |  | 0.1675 |
|  | Current | 48(23.7) | 33(28.4) | 7(21.2) | 8(14.8) |  |
|  | Former | 107(52.6) | 59(50.9) | 20(60.6) | 28(51.9) |  |
|  | Never | 48(23.7) | 24(20.7) | 6(18.2) | 18(33.3) |  |
|  | Pack-year | 18.8±23.7 | 19.4±23.9 | 24.5±24.6 | 14.5±20.3 | 0.1450 |
|  | Biomass exposure | 70(34.8) | 45(39.5) | 12(36.4) | 13(24.1) | 0.1444 |
|  | BMI | 23.3±3.3 | 23.0±2.7 | 22.5±3.5 | 24.7±3.7 | 0.0010 |
|  | Height | 160.2±8.6 | 161.3±8.5 | 160.8±8.1 | 157.9±8.8 | 0.0552 |

BMI ; body mass index

†p values correspond to comparisons between the 3 subgroups using Chi-square test or ANOVA, as appropriate.
